# *Ginkgo biloba L. (Ginkgoaceae)* Leaf Extract Medications From Different Providers Exhibit Differential Functional Effects on Mouse Frontal Cortex Neuronal Networks

**DOI:** 10.3389/fphar.2018.00848

**Published:** 2018-08-03

**Authors:** Benjamin M. Bader, Konstantin Jügelt, Luise Schultz, Olaf H.-U. Schroeder

**Affiliations:** NeuroProof GmbH, Rostock, Germany

**Keywords:** *Ginkgo biloba L. (Ginkgoaceae)*, microelectrode array, functional screening, Alzheimer's disease, *in vitro* model, amyloid beta 1-42

## Abstract

**Background:** Details of the extraction and purification procedure can have a profound impact on the composition of plant-derived extracts, and thus on their efficacy and safety. So far, studies with head-to-head comparison of the pharmacology of *Ginkgo* extracts rendered by different procedures have been rare.

**Objective:** The objective of this study was to explore whether *Ginkgo biloba L. (Ginkgoaceae)* leaf extract medications of various sources protect against amyloid beta toxicity on primary mouse cortex neurons growing on microelectrode arrays, and whether the effects differ between different *Ginkgo* extracts.

**Design:** Our brain-on-chip platform integrates microelectrode array data recorded on neuronal tissue cultures from embryonic mouse cortex. Amyloid beta 42 (Aβ42) and various *Ginkgo* extract preparations were added to the networks *in vitro* before evaluation of electrophysiological parameters by multi-parametric analysis. A Multi-variate data analysis, called Effect Score, was designed to compare effects between different products.

**Results:** The results show that Ginkgo extracts protected against Aβ42-induced electrophysiological alterations. Different *Ginkgo* extracts exhibited different effects. Of note, the reference *Ginkgo biloba L. (Ginkgoaceae)* leaf medication Tebonin had the most pronounced rescuing effect.

**Conclusion:** Here, we show for the first time a side-by-side analysis of a large number of *Ginkgo* medications in a relevant *in vitro* system modeling early functional effects induced by amyloid beta peptides on neuronal transmission and connectivity. *Ginkgo biloba L. (Ginkgoaceae)* leaf extract from different manufactures exhibit differential functional effects in this neural network model. This in-depth analysis of functional phenotypes of neurons cultured on MEAs chips allows identifying optimal plant extract formulations protecting against toxin-induced functional effects *in vitro*.

## Introduction

Drugs and supplements containing various preparations from *Ginkgo biloba L. (Ginkgoaceae)* leaves are widely used in the elderly population (Gafner, 2018). There is a stunning number of various Ginkgo food supplements and medications, containing various leaf preparations or extracts. It is well established that it is not just the plant material that determines the nature, composition and effects of a plant extract, but that it is also highly dependent on the details of the extraction procedure (Itil et al., 1996). Considering the high popularity of Ginkgo extracts, it is an important question how different Ginkgo preparations compare to one another regarding their pharmacological properties. While for one specific extract, called EGb 761, the efficacy and safety for treatment of cognitive impairment, dementia, tinnitus, and vertigo has been demonstrated in multiple clinical studies (von Boetticher, 2011; Gauthier and Schlaefke, 2014; Basta, 2017), such scientific evidence is largely lacking for other Ginkgo products. Therefore, EGb 761 has often been considered the “gold standard” of Ginkgo extract, against which other Ginkgo preparations should be tested (Wohlmuth et al., 2014). EGb 761 decreases blood viscosity, thereby increasing microcirculation (Kellermann and Kloft, 2011); it affects neurotransmission (Yoshitake et al., 2010) and neuroplasticity (Tchantchou et al., 2007, 2009). It prevents oxidative stress (Brunetti et al., 2006; Mohamed and Abd El-Moneim, 2017) and most prominently it protects amyloid beta toxicity (Augustin et al., 2009; Shi et al., 2009, 2010; Tian et al., 2012, 2013; Liu et al., 2015, 2016; Zhang et al., 2015; Scheltens et al., 2016; Wan et al., 2016).

Protective effects against toxic amyloid protein species, especially the Abeta_1−42_ form, are considered to suggest beneficial effects for Alzheimer's disease treatment (Selkoe and Hardy, 2016). Here, we have therefore chosen a cellular model for Amyloid beta toxicity to compare the neuroprotective potential of different Ginkgo preparations.

Functional *in vitro* analysis tools can bridge the gap between morphological and physiological *in vivo* readouts. The use of microelectrode arrays (MEAs) enables the recording of extra-cellular action potentials of a multitude of neurons cultured in a dish and thus elucidatation of the activity characteristics of neuronal networks. This technology has been used extensively for neurotoxicity studies (Gross et al., 1997; Gramowski et al., 2006b, 2011; Johnstone et al., 2010; Defranchi et al., 2011; Hogberg et al., 2011; Novellino et al., 2011; Frega et al., 2012; McConnell et al., 2012; Alloisio et al., 2015; Schultz et al., 2015) but also for functional phenotypic screening of pharmaceutical compounds to elucidate functional modes of action (Gramowski et al., 2004, 2006a; Johnstone et al., 2010; Parenti et al., 2013; Lantz et al., 2014; Hammer et al., 2015; Bader et al., 2017). Also, MEAs analyses have been used for testing food quality or for assessing functional effects of nutrients (Gramowski et al., 2006a; Nicolas et al., 2014; Allio et al., 2015). In the present study, we investigated the functional effects different commercial Ginkgo medications to rescue acute Aβ42-induced effects on primary cortical neuronal networks *in vitro*. To that end, we used neuronal cultures grown on microelectrode arrays. The compound's rescue effect on amyloid beta42 (Aβ42) pre-treated networks was investigated and the functional phenotypic effects assessed by multi-parametric analysis which finally were summarized into a single parameter, the effect score.

## Materials and methods

### Compounds

All test medications were purchased at local or internet-based pharmacies. Stock solutions were generated by grinding the tablets with pestel and mortar and dissolving tablet substance corresponding to 40 mg purified Ginkgo extract/ml in DMSO and diluted 1:2,000 to a final concentration of 20 μg /ml in the the cell culture medium. The stock solution for Tebonin was generated by using “Tebonin 120 mg bei Ohrgeräuschen,” which contains EGb 761. EGb 761® is a dry extract from *G. biloba* leaves (35–67:1), extraction solvent: acetone 60% (w/w). The extract is adjusted to 22.0–27.0% ginkgo flavonoids calculated as ginkgo flavone glycosides and 5.0–7.0% terpene lactones consisting of 2.8–3.4% ginkgolides A, B, C, and 2.6–3.2% bilobalide, and contains <5 ppm ginkgolic acids. Amyloid beta peptides treated with Hexafluorisopropanol (HFIP) were purchased from r-peptide (A-1163-1).

### Ethics

All neural tissue from animal were prepared according to the EU Directive 2010/63/EU on theprotection of animals used for scientific purposes (certification file number 7221.3 ± 2). In thisstudy no animal experiments were performed in accordance with the German Animal Protection §7/2 (Tierschutzgesetz). Time-pregnant animals were purchased and shipped by alicensed animal supplier Charles River, Germany. Animals were stored in a separate room for<24 h after arrival in their transport boxes including food and water equivalent. Animal storage is supervised by an animal welfare officer at NeuroProof GmbH, Germany. Short-term storage of animals in transport boxes is in agreement with Directive (EG) Nr. 1/2005 (Animal safety during transport). The mice were sacrificed by cervical dislocationaccording to the German Animal Protection Act §4.

### Primary cell cultures

As previously published by our group (Gramowski et al., 2011; Gramowski-Voß et al., 2015; Bader et al., 2017) embryonic brain tissue was harvested from E15 NMRI mice (Charles River, Sulzfeld, Germany). Frontal cortex was dissociated enzymatically in DMEM10/10 (10% horse and 10% fetal calf serum) including papain and DNase I, cells were resuspended in DMEM10/10 containing 10 μg/ml laminin (Sigma) at a density of 7.5 × 10^6^ cells/ml, and 150,000 cells were seeded onto each well of 48-well MEA neurochips (Axion Biosystems Inc., Atlanta, GA, USA). Each well contains an array of 16 embedded platinum electrodes resulting in a total of 768 channels. Prior to plating, MEAs were coated with freshly prepared 0.1% polyethyleneimine (PEI, Sigma, 181,978) dissolved in Borate buffer (Fisher Scientific, 28341). Cultures were kept at 37°C in a 10% CO_2_ atmosphere. Half-medium changes were performed twice per week with DMEM containing 10% horse serum. The developing co-cultures were treated on day 5 *in vitro* with 5-fluoro-2′-deoxyuridine to prevent glial proliferation and overgrowth. After 4 weeks in culture, the activity pattern stabilizes and is composed of one coordinated main burst pattern with several coordinated sub-patterns (Gramowski et al., 2004, 2006b). In this study cultures between 28 and 30 div were used. Due to the serum used in the culture medium glia survival is supported in these cultures, and mainly because of proliferation of glia during the first 4 days after plating these neuron-glia co-cultures thus consist of approximately 20% neurons and 80% astrocytes including 1% microglia (Gramowski-Voß et al., 2015).

### Multichannel recordings

Multiwell MEA experiments were performed as described before (Gramowski-Voß et al., 2015). Briefly, recordings were executed with the Maestro recording system by Axion Biosystems Inc. (Atlanta, GA, USA) providing 1,200× amplification, sampling at 12.5 kHz, filtering, and spike detection, delivering whole channel neuronal spike data. Unit separation was performed using Spike Splitter (NeuroProof GmbH, Rostock, Germany) based on different waveform shapes yielding up to 2 units per electrode. For extracellular recordings, MEA cultures were maintained at 37°C and a pH of 7.4 through a continuous filtered and humidified airflow with 10% CO_2_. Recordings were performed in DMEM with 10% horse serum.

### Compound treatment

**Figure 1:**
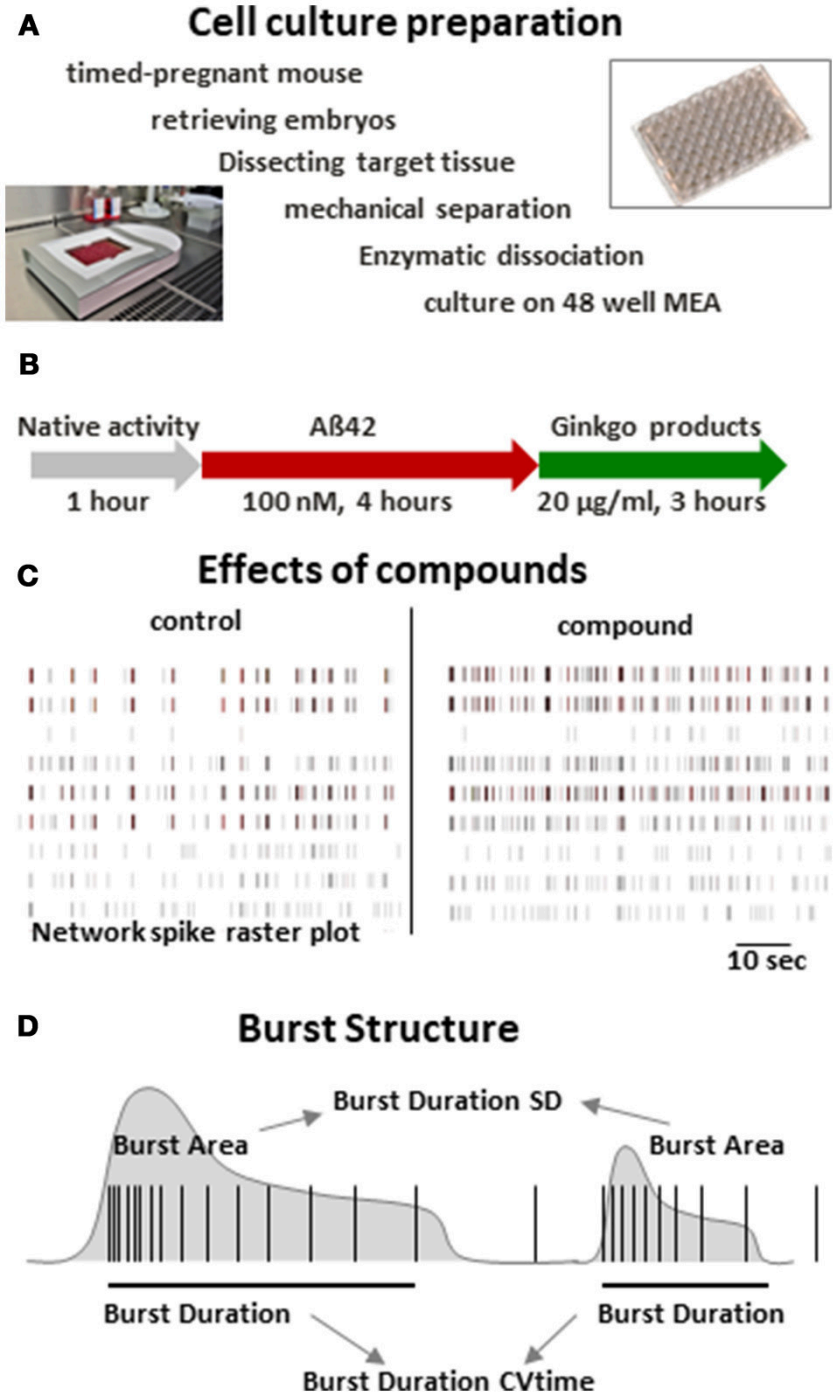
Multi-parametric analysis of cortical neuron network activity. **(A)** Regime of culture preparation of primary mouse neurons. Smaller images: recording setup and 48 well MEA neurochip (Axion Biosystems). **(B)** Time scheme of recording and compound addition. **(C)** Spike raster plots of native cortical activity (control) and cortical activity after acute treatment an excitatory compound illustrating changes in activity patterns. **(D)** Scheme of two simplified bursts outlining some of the parameters extracted from the recordings in this study. SD = standard deviation. CVtime = coefficient of variation over time.

After recording the native activity at 28 days *in vitro*, ultra pure recombinant amyloid beta peptides treated with Hexafluorisopropanol (HFIP) (rPeptide) were added at 100 nM and incubated for 4 h followed by addition of Ginkgo products corresponding to a final concentration of 20 μg purified extract/ml or 0.1% DMSO control which were incubated for 3 h (Figure 1). During the course of the experiment, extracts were prepared by a spearate person than conducting the experiments and data analysis. The test samples were numbered with consecutive numbers, and the experimenter was not aware which sample represented which number.

### Data analysis

A unit represents the activity originating from one recorded neuron. We analyzed the stable activity phase of the last 30 min. Action potentials (spikes) were recorded as spike trains, which are clustered in so-called bursts. Bursts were quantified via direct spike train analysis using the standard interspike interval (ISI) method in NPWaveX (NeuroProof GmbH). Bursts were defined by the following parameters: maximum ISI to start a burst: 40 ms, minimum ISI to end a burst: 200 ms, minimum interval between bursts: 100 ms, minimum duration of burst: 10 ms, and min number of spikes in a burst: 3. Data was normalized against the 4 h Aβ42 treatment phase. Integration of multi-parametric data in the Effect Score including selection of best describing parameters based on their Z'-factor was performed as described earlier (Kozak and Csucs, 2010; Kümmel et al., 2010). For demonstrating Aβ42 effects, data from 75 experiments were pooled in order to include the distribution of the Abeta effect sizes of the complete study into the calculation of the “Effect Score” which was built using 15 parameters with best Z'-factors. Blinded test groups were un-blinded after data analysis thereby reducing the bias.

### Statistical analysis

Time-response effects are shown as mean values ± SEM. Statistical analysis for single-parametric data: unpaired *t*-test with Bonferroni-Holm correction for time series: *p* ≤ 0.05 are represented with ^*^p ≤ 0.01 with ^**^ and *p* ≤ 0.001 with ^***^. For Effect Score measure ANOVA was used followed by Dunnett's test. Number of data points DMSO 5-18, Tebonin 7, Ginkgo-B 7, Ginkgo-C 7, Ginkgo-D 12, Ginkgo-E 8, Ginkgo-F 8.

## Results

### Functional acute effects of amyloid beta 42 (Aβ42)

**Figure 2:**
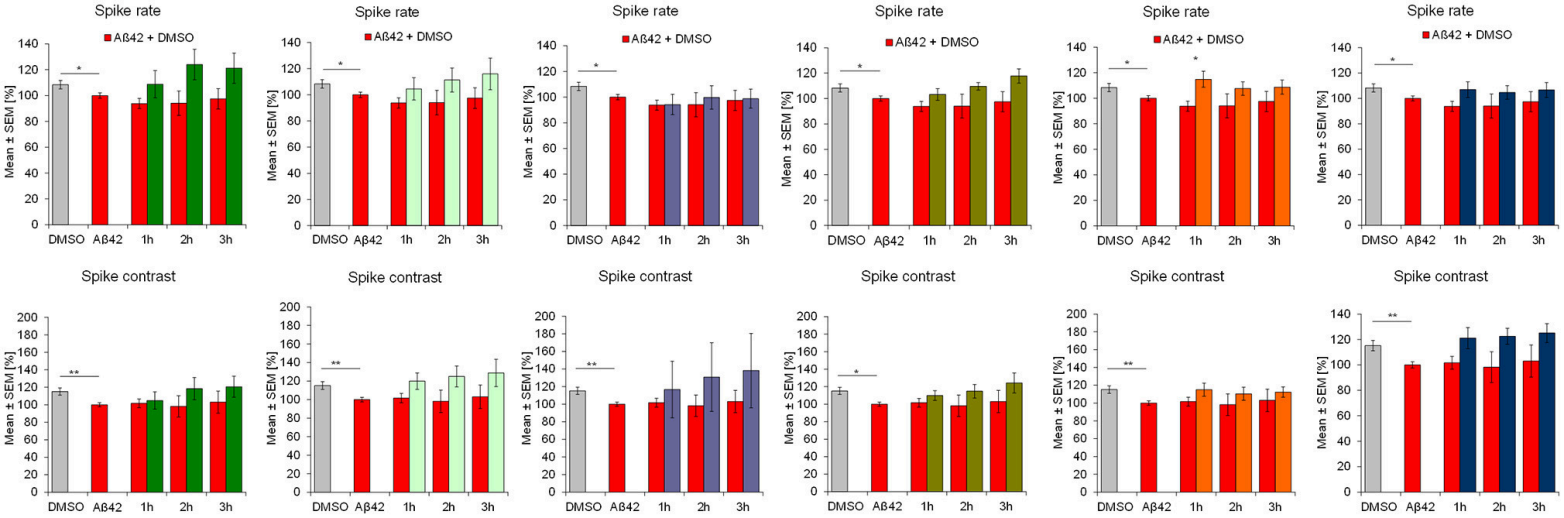
Influence of 100 nM Aβ42 and combination of Aβ42 + 20 μg/ml Ginkgo medications solved in DMSO on **general activity** of cortex network activity *in vitro*. Acute addition of 100 nM Aβ42 induces measurable activity changes of spike rate and spike contrast (reflecting burstiness). Effects increases over time. DMSO control, Aβ42 + DMSO, Aβ42+Tebonin, Aβ42+Ginkgo B, Aβ42 + Ginkgo C, Aβ42 + Ginkgo D, Aβ42 + Ginkgo E, Aβ42 + Ginkgo F. Shown are mean values ± standard error, normalized to native activity. Student's *t*-test with Bonferroni-Holm correction with *p ≤ 0.05.

**Figure 3:**
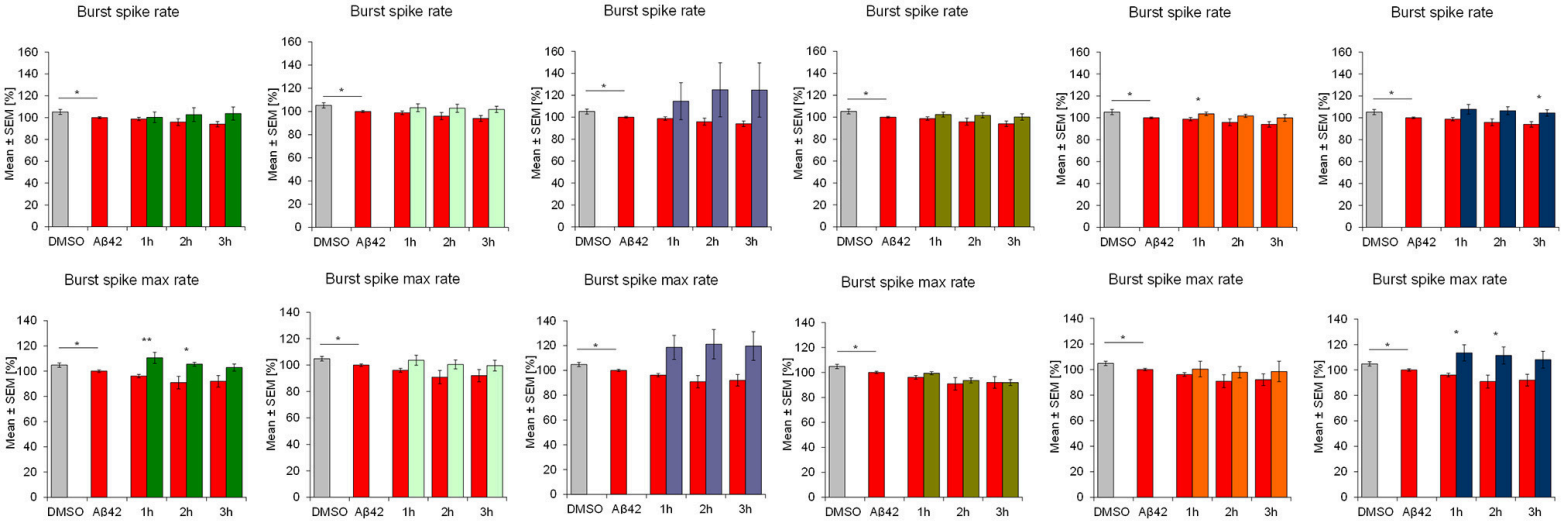
Influence of 100 nM Aβ42 and combination of Aβ42 + 20 μg/ml Ginkgo medications on **burst structure** of cortex network activity *in vitro*. Acute addition of Aβ42 induces measurable activity changes of spike rate inburst and maximal spike rate in burst. DMSO control, Aβ42 + DMSO, Aβ42 + Tebonin, Aβ42 + Ginkgo B, Aβ42 + Ginkgo C, Aβ42 + Ginkgo D, Aβ42 + Ginkgo E, Aβ42 + Ginkgo F. Shown are mean values ± standard error, normalized to native activity. Student's *t*-test with Bonferroni-Holm correction with *p ≤ 0.05.

**Figure 4:**
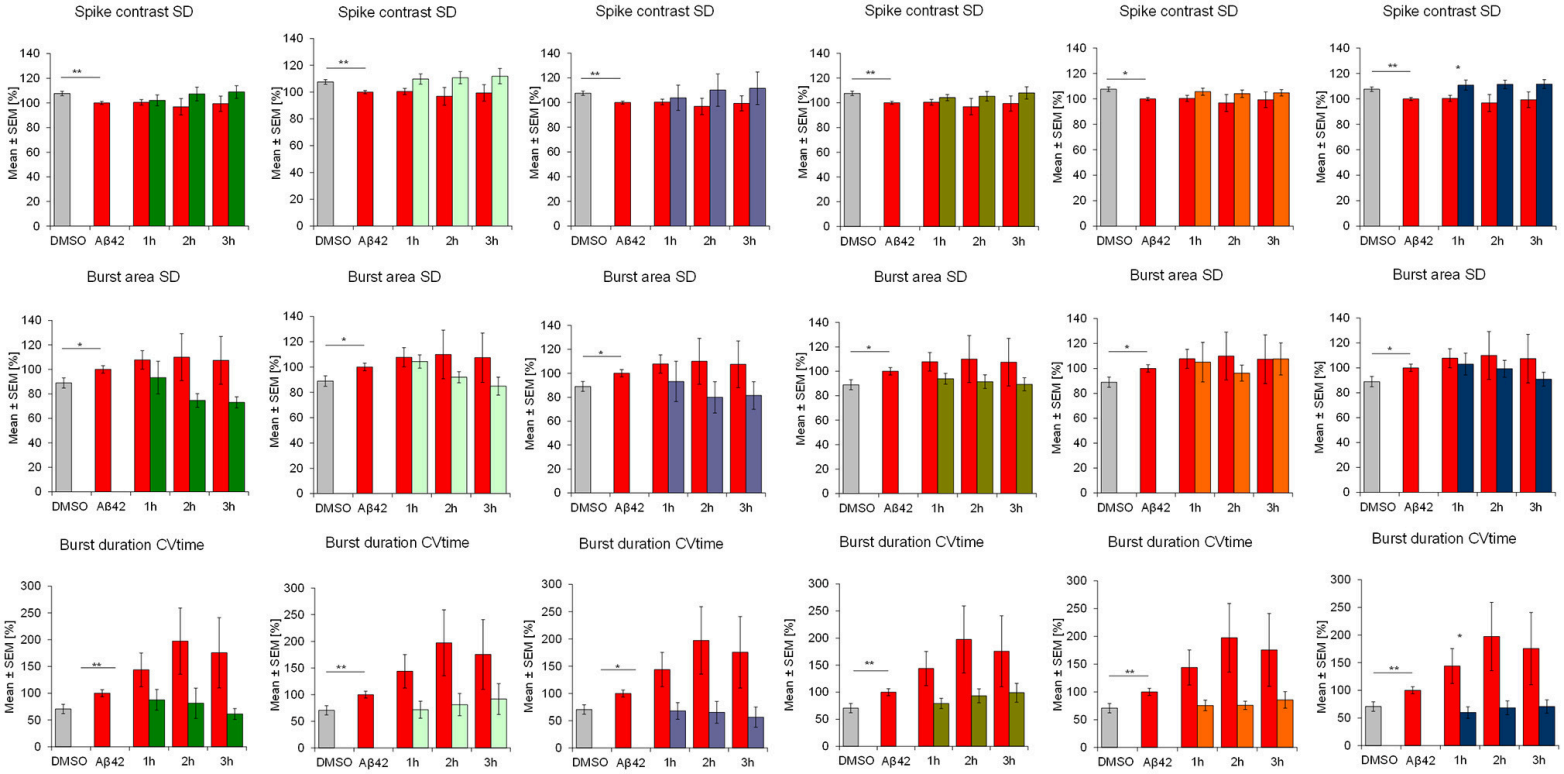
Influence of 100 nM Aβ42 and combination of Aβ42 + 20 μg/ml Ginkgo medications on **regularity** of cortex network activity *in vitro*. Acute addition of Aβ42 induces measurable activity changes of spike contrast SD, burst area SD and burst duration CVtime reflecting the variation over time as an indicator for regularity. DMSO control, Aβ42 + DMSO, Aβ42 + Tebonin, Aβ42 + Ginkgo B, Aβ42 + Ginkgo C, Aβ42 + Ginkgo D, Aβ42 + Ginkgo E, Aβ42 + Ginkgo F. Shown are mean values ± standard error, normalized to native activity. Student's *t*-test with Bonferroni-Holm correction with *p ≤ 0.05.

Aβ42 addition acutely affected the activity of frontal cortex neurons which is seen in multiple parameters. After 4 h of incubation with 100 nM synthethic HFIP-treated Aβ42, the overall spiking activity was slightly but statistically significantly reduced which was accompanied by reduction of “burstiness” indicated by the spike contrast (Figure 2). Aβ42 also affected the burst structure shown by reduced spike rate in burst (burst spike rate) and the maximal spike rate in bursts (burst spike max rate, Figure 3). The calculation of standard deviation (SD) and coefficient of variation over time (CVtime) of general activity or burst structure parameters reflect the regularity of periodic events, while increased values reflect increased variation and thus, lower regularity. Aβ42 increased burst structure variation (burst area SD, burst spike number CVtime, burst duration CVtime, Figure 4) indicating a subtle increase of irregularity of the normally regular cortex activity pattern. The neuronal phenotype affected by Aβ42 was used as the baseline to screen compound-induced effects.

### Rescue of Aβ42 effects by *Ginkgo biloba l. (Ginkgoaceae)* exctract tebonin

The reference compound Tebonin showed a rescue of Aβ42-mediated effects indicated by the return to activity levels of DMSO-treated networks within 3 h post-compound treatment. Noteworthy, some parameters showed a time-dependent increase of Aβ42 effects (e.g., burst duration CVtime). Tebonin stopped these effects after 3 h and reverted the activity values toward control condition. Some parameters (e.g., burst spike max rate) were instantly rescued within 1 h. All parameters had in common that Tebonin shifted the activity levels toward control condition, therefore, representing a rescue effect.

This feature of rescuing acute functional Aβ42 effects was also observed for other commercially available *Ginkgo biloba L. (Ginkgoaceae)* medications (ginkgo-B, -C, -D, -E, F) tested in this experimental regime. However, differences of rescue effects were observed: e.g., Ginkgo-C showed no rescue effects on spike rate (Figure 2); Ginkgo-D had no effects on burst spike max rate and smaller effects on burst duration, CVtime and burst spike number CVtime (Figures 3, 4); Ginkgo-E had no effects on burst area SD (Figure 4).

This complex combination of different reactions complicates the comparison of rescue effects between the different compounds. Therefore, we used a linear combination of multiple parameters to enable ranking of rescue effects with one value.

### Integration of multi-parametric data and ranking of rescue effects

To compare the rescue effects of acute functional Aβ42 effects between the test substances and positive control Tebonin, the 15 best-describing parameters from all 204 calculated were selected based on their Z'-factor using the method described earlier (Kozak and Csucs, 2010; Kümmel et al., 2010).

**Figure 5:**
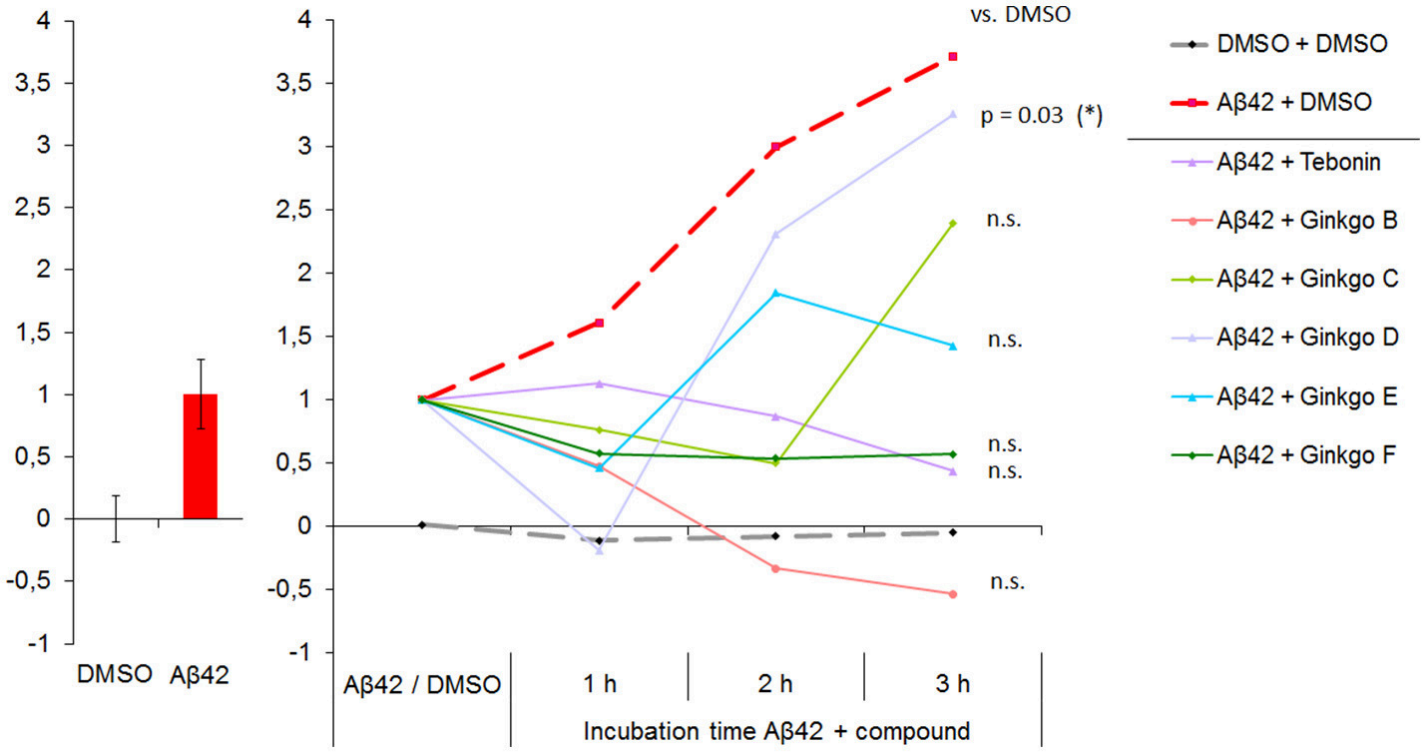
Multi-parametric Aβ42 effects are integrated and presented as “NP Effect Score”. **Left**: NP Effect Score for DMSO and Abeta normalized to “0” and “1”, respectively. 15 best-describing parameters (partly including those showin in Figure 2) were integrated in the Effect Score. **Right**: NP Effect Score for all test compounds. Starting point is the intrinsic Aβ42 effect (set to 1). DMSO control is stable over time. The 3 h time point represents the time of highest resolution and thus the optimal time point for ranking compound efficacies to rescue Aβ42 effects toward DMSO control conditions. N-DMSO = 18, N-Aβ42 = 75. At 3 h time point: ANOVA with *p* ≤ 0.033, Dunnett's test with *p ≤ 0.05.

The result of this parameter integration of Aβ42 effects compared to DMSO control was termed the Effect Score. As described in the methods section, to calculate the Effect Score, values after 4 h Aβ42 treatment were set to 1, and the DMSO control was set to 0 (Figure 5). Using this calibration, the Effect Score was also calculated for 1, 2 and 3 h post-Aβ42 addition which showed a continuous increase from 1 to 3 h. The datasets of the Aβ42 + DMSO and Aβ42 + Ginkgo test compound combinations were then integrated using the Aβ42/DMSO calibration.

On the single parameter level, the Aβ42 + DMSO group showed an increase of Aβ42-specific parameters, indicating a time-dependent effect. In agreement, on the integrated parameter levels the Effect Score also increased over time, but showed a more than 3.5–fold increase of the Effect Score within 3 h which is not as obvious when focusing on single parameters. The DMSO Effect Score maintained stable over time (Figure 5). Three hours after addition, Aβ42 showed the highest deviation from DMSO effects. This time point was therefore used to rank the test compounds for their rescue capacity. The rescue capacity is defined by the difference to DMSO which can be either below or above 0. Values below 0 represent compound effects which change parameter levels beyond DMSO levels. The effect score values of all tested compounds are listed in Table 1. Tebonin showed the strongest rescue directly followed by Ginkgo-B, which showed a negative Effect Score. Ginkgo-D showed a transient rescue at 1 h but lost the rescue efficacy thereafter, demonstrating the least rescuing effect at the 3 h time point.

**Table 1:** Rescue efficacy of different Ginkgo medications.

	**relative distance from DMSO control [%]**	**rank**
Tebonin	12	1
Ginkgo B	14^*^	2
Ginkgo F	15	3
Ginkgo E	38	4
Ginkgo C	64	5
Ginkgo D	87	6

The reference medication Tebonin showed with 12% the highest rescue indicated by relative disctance from DMSO [%].

* *Ginkgo B showed a negative Effect Score value (compare Figure 5) highlighting the importance for using the distance from control as the most relevant rescue efficacy measure*.

## Discussion

Even when two extracts are derived from the same plant species, their composition, efficacy and tolerability can vary considerably. There is a large number of *Ginkgo* food supplements and medications on the market; their individual composition and effects are determined by the kind and quality of the plant material and, importantly, also by the extraction procedure (Itil et al., 1996). The most extensively examined extract, EGb 761, supports neurotransmission (Yoshitake et al., 2010) and neuroplasticity (Tchantchou et al., 2007, 2009) and protects against amyloid beta toxicity (Augustin et al., 2009; Shi et al., 2009; Tian et al., 2013; Liu et al., 2015; Scheltens et al., 2016; Wan et al., 2016) which includes prevention of oxidative stress (Brunetti et al., 2004, 2006; Mohamed and Abd El-Moneim, 2017) and is involved in improving neuro degeneration-induced downregulation of monoamine signaling (Chen et al., 2007; Ferrante et al., 2017). Protection against toxic amyloid protein species, especially the 1–42 forms, suggests potential beneficial effects for Alzheimer's disease (AD) treatment (Selkoe and Hardy, 2016). Therefore, a functional *in vitro* test system to analyze compound rescue efficacies against Aβ42-induced effects is valuable for evaluating treatments for AD. Functional electrophysiological readouts may be optimal for detecting early pathophysiological events which trigger cytotoxicity later on. Thus, a balance between detectable functional effects without pronounced cytotoxic influence is desirable. To establish AD-relevant *in vitro* models, primary neurons from adult diseased animals are the superior choice but difficult to culture for extended periods with goal to obtain a spontaneously active neuronal network. These neuronal networks can be formed using embryonic culture within days. Thus, brain slices and embryonic cultures are used for *in vitro* functional electrophysiological studies on the effects of Aβ which mostly are conducted using the patch clamp method (Lambert et al., 1998; Jhamandas et al., 2001; Gureviciene et al., 2003). However, *in vitro* microelectrode array (MEA) cell culture systems with primary embryonic rodent neuron cultures also provide a means to detect acute and chronic functional effects of Aβ42 peptides at sub-cytotoxic concentrations. Noteworthy, embryonic *in vitro* cultures mature over time and stabilize after 21 days *in vitro* (div) (Ito et al., 2013). We and others observed that bursting activity patterns of cortical neurons reach a plateau phase between 21 and 28 div and peak around 28 div (Chiappalone et al., 2006; Wagenaar et al., 2006). Therefore, we used 28 div cultures for this study. MEA experiments with 100 nM Aβ42–the concentration used in this study–showed specific effects including a reduction of general spiking activity, bursting strength and synaptic connectivity when applied to 28 div cortical neurons (Kirazov et al., 2008). This concentration was shown to be not accompanied by dramatic cytotoxic effects (Varghese et al., 2010). At concentrations above 5 μM, however, Aβ42 effects were shown to induce a more significant inhibition of network activity and connectivity which occurs within 4 h post-treatment and exhibits a time-dependent effect within 24 h (Kirazov et al., 2008; Charkhkar et al., 2015). These 5 μM tests were accompanied by significant cyto-toxic effects (Varghese et al., 2010; Charkhkar et al., 2015), which we wanted to avoid in the experiments described here. Therefore, we acutely treated mouse frontal cortex neurons with 100 nM human recombinant Aβ42 peptides and quantified the MEA readouts by multi-variate analyses. Several brain regions including hippocampus, hypothalamus and frontal cortex are affected in AD (Magalingam et al., 2018), thus, we selected frontal cortex cultures after 28 div for this study, also because in our hands cortical neurons showed a more assay-relevant reproducible phenotype compared to e.g., hippocampus (not shown). We show that 100 nM Aβ42-induced acute inhibitory effects increase in a time-dependent manner up to 7 h. We thereby extend previous reports (Varghese et al., 2010; Charkhkar et al., 2015) in a higher temporal resolution. Four h after Aβ42 was applied, different Ginkgo medications were added to investigate and compare their rescue efficacy. We show that the reference compound Tebonin reverted Aβ42-induced parameter changes toward control condition. Rescue was observed at different parameters within 3 h after Tebonin addition. The other tested Ginkgo products also showed rescue effects. Noteworthy, the parameter-specific rescue effects differed between the Ginkgo products, thereby complicating the comparison between the groups. For that reason we used a linear parameter combination (Kozak and Csucs, 2010; Kümmel et al., 2010) to integrate the Aβ42-affected parameters. This parameter, the Effect Score, allowed comparing the rescue effects between the *Ginkgo* products. One compound (i.e., Ginkgo-B) showed an Effect score below 0, suggesting an overshooting beyond the control profile. As the optimal Effect score is defined by DMSO control of 0, we defined the efficacy to rescue Aβ42-induced effects by the distance to the DMSO control functional profile which can be either below or above 0. In summary, the effect score values (Table 1) show that the reference medication Tebonin induced the strongest rescue with 12% distance to DMSO, directly followed by Ginkgo-B, which showed a negative Effect Score of −14% and Ginkgo-F with 15%. Ginkgo-C, -D and -E showed a lower rescue effects. The negative Effect Score value of Ginkgo B indicates that compounds can also affect the parametric shift beyond control levels and over compensate. The goal, however, was to find the Aβ42+compound mixture which resulted in a functional phenotype as similar to DMSO as possible which was the Aβ42+Tebonin combination.

Tebonin contains the Ginkgo extract EGb 761 was shown to be effective for treatment of cognitive impairment and dementia (von Boetticher, 2011; Gauthier and Schlaefke, 2014) and for affecting neurotransmission (Yoshitake et al., 2010) and neuroplasticity (Tchantchou et al., 2007, 2009). Here, for the first time, we systematically compare different commercially available Ginkgo products in one experimental *in vitro* approach and show that the different Ginkgo products with different extraction procedures (Itil et al., 1996) exhibit different functional effects.

## Author contributions

BB and OS designed research; LS performed experiments, LS, BB, and KJ analyzed data; BB made the figures, BB wrote the paper.

### Conflict of interest statement

This study was partly sponsored by Dr. Willmar Schwabe GmbH & Co KG, Karlsruhe, Germany. OS, KJ, LS, and BB are emplyees of NeuroProof. OS holds shares of NeuroProof.
